# Supplementary material for: Patterns of Intron Gain and Loss in Fungi
Source: PLoS Biol. 2004 Nov 30;2(12):e422. doi: 10.1371/journal.pbio.0020422 (PMC532390; doi:10.1371/journal.pbio.0020422)
Supplement: Table S1 — Also available at http://genes.mit.edu/NielsenEtAl/. (4.3 MB ZIP). [file pbio.0020422.st001.zip › NielsenEtAl/html/847.html]

AN1136.1.NCU06022.1.MG07783.1.FG05701.1


```
 CLUSTAL W (1.82) Multiple Sequence Alignments - Introns Inserted


Sequence 1: MG07783.1	352 aa
Sequence 2: FG05701.1	296 aa
Sequence 3: NCU06022.1	340 aa
Sequence 4: AN1136.1	315 aa
Alignment Length: 368 aa
Number Identitical Residues: 156 aa
Alignment Score (without introns) 8083


MG07783.1 	MADTTLNDKELQEAYDFAVQLGKDAGAMLMDAARARFG--------TSAAQKSVEKERS1
NCU06022.1	MTSRTTTATELDEIYTFAVQLGKDAGNLLMEAARLRFSNNNANHDKESTTQEFTEKDSA~
FG05701.1 	-----MDSQQLSEIYAFAVQLGKDAGDMLMAAAQRRIDG----HGTSSTTVSYVEKENS~
AN1136.1  	-MDCPIPQTELDEIYAFATDLARKAGQLLLERVNDRNS-----------EQVYAEKENA~
          	  . .    :*.* * **.:*.:.** :*:  .. * .               .**: : 

MG07783.1 	NKYHGYGLAVDTPAGPPGSLSAVDIVTETDFK~VEEFIRTTIAKKYPS~HG2FIGEETYS
NCU06022.1	----------------------VDIVTQTDED~VEAFIKSAINTRYPS~HD~FIGEETYA
FG05701.1 	----------------------VDLVTKTDND1VENFIRTTVASKYPE~HG2FLGEESYS
AN1136.1  	----------------------VDLVTQTD--~-------------EA1HK~FLGEESYA
          	                      **:**:**                   *  *:***:*:

MG07783.1 	AG--ASRDYLVT-SSPTWIVDPLDG~TVNFTHLFPMFCVSIGLVVDHDPVVGVINAPFAN
NCU06022.1	KSSQSTRPYLVTHTTPTWVVDPLDG~TVNYTHLFPMFCVSIAFLVDGTPVIGVICAPMLG
FG05701.1 	TG--ASRTYLVD-DKPTWVVDPLDG~TVNFTHLFPMFCVSIAFVVKGVPVIGVINAPFLR
AN1136.1  	KG--QSREYLID-EQPTWCVDPLDG1TVNFTHAFPMFCVSIGFIVNHYPVIGVIYAPMLN
          	 .   :* **:    *** ****** ***:** ********.::*.  **:*** **:  

MG07783.1 	QFFSACRGRGAWLNETVPLPLVRD---SPLPPLAPKGCIFSCEWGKDRRDRPDGNLHRKV
NCU06022.1	QLFTACKGRGAWLNETQRLPLVRQ----PMPKSAPGGCVFSCEWGKDRKDRPEGNLYRKV
FG05701.1 	QFFSSCTGQGAWLNETQRLPLIRNP-IPPMPANAPSACTFSCEWGKDRRDIPDGNLSRKI
AN1136.1  	QLFSSCLNRGAWLNEMQQLPLIRKPSIPPLPATAPSKCIFACEWGKDRRDIPDGTLQRKI
          	*:*::* .:******   ***:*..: .*:*  **  * *:*******:* *:*.* **:

MG07783.1 	ETFVNMATELGGRGGRGGMVHGIRSLGS~ATLDLAYTAMGSFDIWWEGGCWEW2DVAAGI
NCU06022.1	ESFVNMAAEVGGRGGKGGMVHGVRSLGS2ATLDLAYTAMGSFDIWWEGGCWEW2DVAAGI
FG05701.1 	ESFVNLASERNGRGGKGGMVHGVRSLGS2ATLDLAYVAMGSLDIWWEGGCWEW2DVAAGF
AN1136.1  	ESFVNMAAERGSRGGKGGMVHGVRSLGS~ATMDLAYTAMGSVDIWWEGGCWEW2DVAAGI
          	*:***:*:* ..***:******:***** **:****.****.*********** *****:

MG07783.1 	AILKEAGGLVTTATPPSDPETADIQDARLGSRLYLGIR2PAGPSEGETARQAQERVVREV
NCU06022.1	AILQEAGGLITSANPPEDWATAEIPDVKLGSRLYLVVR~PAGPSEGETAREGQERTIREV
FG05701.1 	AILEEAGGLVTTANPPNDPETAEIARAHLGGRLYLAIR~---------------------
AN1136.1  	AILLEAGGLVTAANPPED-IEGPIEPVKLGSRLYLAIR2PAGPSETETGRETQERTVREV
          	*** *****:*:*.**.*   . *  .:**.**** :* .:..:. .:. . ..    . 

MG07783.1 	WKRVKGLDYSRPGA--
NCU06022.1	WRRVRALDYTRPGA--
FG05701.1 	----------------
AN1136.1  	WRRVRQLDYERPTRQS
          	       .   .  .:
```
